# Primary prevention of childhood obesity through counselling sessions at Swedish child health centres: design, methods and baseline sample characteristics of the PRIMROSE cluster-randomised trial

**DOI:** 10.1186/1471-2458-14-335

**Published:** 2014-04-09

**Authors:** Nora Döring, Lena M Hansson, Elina Scheers Andersson, Benjamin Bohman, Maria Westin, Margaretha Magnusson, Christel Larsson, Elinor Sundblom, Mikaela Willmer, Margareta Blennow, Berit L Heitmann, Lars Forsberg, Sanna Wallin, Per Tynelius, Ata Ghaderi, Finn Rasmussen

**Affiliations:** 1 Department of Public Health Sciences, Karolinska Institutet, Child and Adolescent Public Health Epidemiology, Tomtebodavägen 18A, Stockholm SE-171 77, Sweden; 2 Department of Clinical Neuroscience, Karolinska Institutet, Division of Psychology, Stockholm, Sweden; 3 Department of Women’s and Children’s Health, Uppsala University, Uppsala, Sweden; 4 Department of Food and Nutrition and Sport Science, University of Gothenburg, Gothenburg, Sweden; 5 Department of Food and Nutrition, Umeå University, Umeå, Sweden; 6 Centre for Epidemiology and Community Medicine, Stockholm County Council, Health Care Services, Stockholm, Sweden; 7 Department of Clinical Science and Education, Child Health Services, Södersjukhuset, Stockholm, Sweden; 8 Research Unit for Dietary Studies, Institute of Preventive Medicine, Copenhagen University Hospital, Copenhagen, Denmark; 9 The Boden Institute of Obesity, Nutrition, Exercise & Eating Disorder, University of Sydney, Sydney, Australia; 10 National Institute of Public Health, University of Southern Denmark, Odense, Denmark; 11 Department of Clinical Neuroscience, Karolinska Institutet, Centre of Psychiatry Research, Stockholm, Sweden

**Keywords:** Childhood obesity, Primary prevention, Motivational interviewing, Primary care setting, Intervention study

## Abstract

**Background:**

Childhood obesity is a growing concern in Sweden. Children with overweight and obesity run a high risk of becoming obese as adults, and are likely to develop comorbidities. Despite the immense demand, there is still a lack of evidence-based comprehensive prevention programmes targeting pre-school children and their families in primary health care settings. The aims are to describe the design and methodology of the PRIMROSE cluster-randomised controlled trial, assess the relative validity of a food frequency questionnaire, and describe the baseline characteristics of the eligible young children and their mothers.

**Methods/Design:**

The PRIMROSE trial targets first-time parents and their children at Swedish child health centres (CHC) in eight counties in Sweden. Randomisation is conducted at the CHC unit level. CHC nurses employed at the participating CHC received training in carrying out the intervention alongside their provision of regular services. The intervention programme, starting when the child is 8-9 months of age and ending at age 4, is based on social cognitive theory and employs motivational interviewing. Primary outcomes are children’s body mass index and waist circumference at four years. Secondary outcomes are children’s and mothers’ eating habits (assessed by a food frequency questionnaire), and children’s and mothers’ physical activity (measured by accelerometer and a validated questionnaire), and mothers’ body mass index and waist circumference.

**Discussion:**

The on-going population-based PRIMROSE trial, which targets childhood obesity, is embedded in the regular national (routine) preventive child health services that are available free-of-charge to all young families in Sweden. Of the participants (n = 1369), 489 intervention and 550 control mothers (75.9%) responded to the validated physical activity and food frequency questionnaire at baseline (i.e., before the first intervention session, or, for children in the control group, before they reached 10 months of age). The food frequency questionnaire showed acceptable relative validity when compared with an 8-day food diary. We are not aware of any previous RCT, concerned with the primary prevention of childhood obesity through sessions at CHC that addresses healthy eating habits and physical activity in the context of a routine child health services programme.

**Trial registration:**

ISRCTN16991919

## Background

Childhood obesity is a growing concern worldwide, and its prevention is one of the key elements in current public health strategies in many countries, including Sweden. Children with overweight and/or obesity run an increased risk of becoming obese as adults, are likely to develop comorbidities, such as type-2 diabetes and coronary heart disease (CHD), and are at higher risk of premature death [1,2]. In comparison with many other Western countries, the obesity and overweight trends in Sweden are less pronounced, and there are signs of stabilisation in the overweight trend in certain sub-groups [3]. Nonetheless, there is an indication that the prevalence of overweight and obesity is rising in the Swedish adult population. The most recent estimates of population prevalence of adult overweight (35.1%) and obesity (11.2%) are at their highest levels in recent history [4]. Among 4-year-old children, prevalence rates seem to level off, but remain an important public health problem, since 14% of boys and 19% of girls are overweight and/or obese in Sweden [5] with prevalence at its highest among socially disadvantaged groups [6].

Eating and physical activity (PA) habits are established early in life, and become less malleable with age [7]. Parental practices, such as feeding styles and making (un)healthy food products available at home, and also parents’ nutritional knowledge and health behaviours are of major importance for how young children’s eating and PA habits emerge [8,9]. Primary preventive efforts are likely to have optimal effects if started in early childhood, and if designed to include parents [10,11].

A recent systematic review of prevention in the field of childhood obesity found some evidence that intervention programmes, mainly in schools and day-care settings, aimed at promoting healthy eating habits and PA, can prevent childhood obesity [12], but other systematic reviews have shown less or no preventive effect [10,13,14]. Especially for children aged 0-5, the evidence base remains weak. Waters et al. [12] examined eight intervention studies targeting children 0-5. However, in seven of these studies, the children were already at least four years of age at baseline. Thus, while the need for early childhood obesity prevention is widely recognised, there is still a lack of evidence concerning the effects of interventions targeting preschool children in settings other than school and day-care, and in particular in (child) health care. Another recent systematic review investigated the effects of different types of counselling with parents of children 0-5 years of age [15]. This review suggests that counselling interventions based on different health behaviour theories, particularly social cognitive theory (SCT), that are structured and use patient-centred dialogue over several sessions, seem to increase young children’s fruit and vegetable intake.

In order to obtain a structured prevention programme based on a theory in harmony with a client-centred counselling approach, we chose SCT as our theoretical framework [16,17]. A core element in SCT is dynamic interplay between interpersonal factors, behavioural factors, and environmental factors. In intervention research, applications of SCT are characterised by efforts to increase self-efficacy, i.e., people’s beliefs in their capacity to accomplish behaviour change in a specific domain of functioning, e.g., healthy eating [18]. An efficient and brief method for preparing people for behaviour change in a client-centred setting is motivational interviewing (MI). MI is a collaborative, client-centred counselling style for promoting behaviour change [19]. Eliciting and reinforcing motivation for and commitment to behavioural change are central to the method [19-21]. In previous research, MI has proved to be effective in changing unhealthy dietary and PA habits [21]. Whereas SCT is a coherent theory that conceptualises human motivation and behaviour, MI is a clinical method for communicating with patients or clients.

Finally, we chose Swedish child health care centres (CHCs) as our intervention arena, with CHC nurses as counsellors, since this context would have high ecological validity for future implementation of the programme.

Due to the health and social consequences for individuals and their families, and also because of the health economic consequences of overweight and obesity for society, decision-makers are in great need of evidence-based and cost-effective primary prevention programmes that actually work.

### Aim

This paper presents the study design and methodology of the PRIMROSE trial, including the relative validation of a food frequency questionnaire (FFQ), and also describes the baseline characteristics of the participating young children and their mothers.

PRIMROSE is a population-based trial, designed, in a primary health care setting, to promote healthy eating and PA behaviours among pre-school children (9-48 months of age) and their parents. The overarching goal is to evaluate an intervention intended to stabilise and reverse obesity prevalence among preschool children in Sweden. The specific aims of the PRIMROSE trial are to answer the following questions:

1) Does a structured and specific intervention programme based on SCT and MI have effects on preschool children’s and their parents’ eating habits, physical activity patterns, and the prevalence of overweight or obesity?

2) Is such an intervention cost-effective? That is, how large are the additional costs of the intervention in relation to its health effects in comparison with care as usual?

## Design and methods

This cluster-randomised intervention trial is coordinated by a team at the Karolinska Institutet in Stockholm (Sweden) in collaboration with international experts from the universities of Uppsala (Sweden), Copenhagen (Denmark), and Gothenburg (Sweden). Approval (2006/525-31/2) was obtained from the *Regionala Etikprövningsnämnden* Stockholm (The Ethical Review Board Stockholm), and the trial has been registered (ISRCTN16991919).

### Setting

The trial operates at Swedish CHCs in eight counties. These centres are a well-established part of Swedish primary health care services, which are responsible for health promotion and the health surveillance of children from birth up to school age. Such surveillance is most often achieved by individual family counselling, but can also include group sessions with other parents. As well as regular health check-ups and vaccinations, parents are offered advice on their young children’s health and development. However, the advice and guidance received by parents are mainly about breastfeeding, child growth, vaccinations, prevention of accidents, etc., with minimal time spent on health behaviours [22]. The services are free of charge and attended by nearly all families with young children, irrespective of social position or ethnicity [23]. The PRIMROSE trial is embedded within these regular Swedish child health services, and conducted under normal working circumstances.

### Participants

The eight counties (Stockholm, Uppsala, Södermanland, Örebro, Gävleborg, Västernorrland, Västmanland, and Jämtland) were selected on the basis of their relative closeness to Stockholm and/or accessibility. Approximately 37% of the Swedish population lives in these eight counties. When recruitment started in 2008, 1007 CHC nurses were employed in the eight counties and were invited to participate. Of these nurses, 129 (12.8%) agreed to participate in the trial. Before recruitment of a CHC nurse to the PRIMROSE trial, a contract regulating mutual responsibilities was signed between the administrative head of the CHC in question and the principal investigator (FR).

To assess any potential selection bias, all nurses working at CHCs in the participating counties were requested to respond to a questionnaire concerning their education and training, as well as the number of children for whom they were professionally responsible. Of the 1007 nurses, 701 (69.9%) responded to the questionnaire. At baseline, trial nurses had a mean age of 48.3 (SD = 9.0) years and had worked as a CHC nurse for an average of 10.6 (SD = 8.3) years. A majority of trial nurses (68%) had specialist training in primary health care, while 30% had a specialist education in child and adolescent health (compared with 74% and 26%, respectively, of all CHC nurses in the study area). Among the trial nurses, 63% had already received some type of short MI training prior to enrolment as a trial nurse (compared with 56% among all nurses). The number of preschool children for whom a nurse was responsible did not differ between trial nurses (mean = 45.2, SD = 21.8) and non-trial nurses from the same region (mean = 49.0, SD = 22.3).

**Figure 1:**
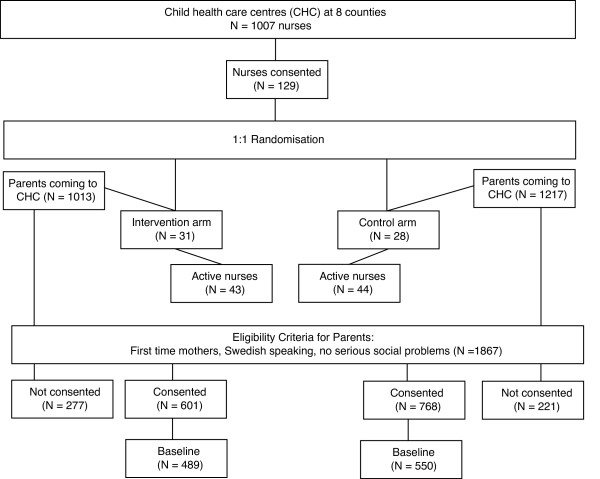
Flow chart.

First-time parents receiving preventive services at a participating CHC (n = 2230) were asked by the trial nurses whether they wished to participate when their children were 5-6 months of age. Families were excluded if they did not speak Swedish (n = 172), were about to change their CHC (n = 49), or had severe social family problems making it unethical to ask them to participate (n = 67), or for other (non-reported) reasons (n = 75). A total of 1867 families were eligible for participation, of which 1369 (73.3%) agreed to participate in the trial (see Figure 1).

### Allocation

Cluster randomisation was performed at CHC-unit level (n = 59), where a unit consists of one or more CHCs (n = 75) belonging to the same local primary health care administration. After obtaining informed consent, participating CHC units were randomly allocated to either the intervention arm (n = 31) or the control arm (n = 28). Within each county, 50% of the CHC units were chosen for the intervention arm (n) and 50% for the control arm (m) whenever the total number of CHC units was even (i.e., n = m). If the number of CHC units was uneven, >50% of them were chosen for the intervention arm (i.e., n-1 = m). Randomisation was performed on 10 different occasions, from March 2008 to January 2010.

### Training programme for intervention nurses including motivational interviewing

The nurses employed at the intervention CHCs (n = 43) took part in a five-day course in SCT and MI, and in nutrition and PA, given by our team [24,25]. The course included role plays of encounters between nurses and parents, and a didactic presentation of the basic principles of behaviour change given by a psychologist. The nutrition component covered healthy eating patterns, food choices, and parental feeding practices. Additionally, nurses were guided by a comprehensive, structured manual written by our team. As soon as the nurses had finished the course, they were encouraged to start practising MI in their routine work at the CHCs, and as part of the training programme, asked to record four sessions with families attending their CHC but not participating in the PRIMROSE trial. At the next step, each intervention nurse was asked to record eight additional sessions with families randomised from all the available intervention families. Thus, twelve sessions with different families were audio recorded. Intervention nurses received individual structured and systematic feedback on the nine first recordings in supervision sessions given by experienced MI experts by telephone. The first and the 5^th^ to 9^th^ of these sessions were evaluated for MI proficiency by professional coders at the Motivational Interviewing Coding Laboratory at Karolinska Institute, Stockholm, Sweden in accordance with the Motivational Interviewing Treatment Integrity [26] in order to provide the MI supervisors with an independent and reliable base for their supervision. In addition, the last three sessions were MITI coded, but no supervision was offered. All nine MITI coded sessions will be used to test the integrity of the MI methodology. The nurses employed at the control CHC (n = 44) were offered neither the course nor the supervision. They simply followed the traditional child health surveillance programme.

### The intervention

The on-going cluster-randomised controlled trial compares regular CHC services (“care as usual”) with participation in the intervention embedded in regular CHC services. Based on SCT and by applying MI principles and strategies, nurses assist parents to change their own health behaviours and to promote healthy dietary and PA behaviours in their children [19,21]. In addition, the intervention attempts to make parents aware of the importance of the context they create for their child with regard to eating and PA habits (e.g., accessibility of fruit and healthy food choices, active physical play, etc.). For each family, the intervention encompasses nine sessions with CHC nurses on child health behaviours (eating habits and PA).

Parents belonging to intervention CHCs are offered their first individual session when their child is approximately nine to ten months old. Group sessions with participating families follow, approximately one month after the first session. When the children are 1.5 years, 2 years, 3 years, and 4 years old, the parents are offered individual sessions in conjunction with regular CHC services. During these sessions, parents, together with the nurses, formulate goals with regard to PA and healthy eating habits. The nurses (together with the parents) write these goals down on a goal formulation sheet, which is available in the intervention manual along with user instructions. Furthermore, when the children are 30 and 42 months old, the families are called by their nurse for discussion of their goals for behaviour change (see Table 1). By contrast, families belonging to the control CHCs are only offered traditional health check-ups at similar ages, with no systematic efforts made to elicit and reinforce motivation for and commitment to behaviour change.

**Table 1:** Overview of the intervention sessions

	**Type**	**Duration (min)**	**Age of child (months)**	**Content**
1	Individual session	90	9-10	• Information is given to parents concerning the intervention
				• Create motivation
				• Introduce the idea of parents as role models for healthy eating and physical activity
2	Group sessions	90-120	11	• Repeat information from the previous session
				• Give presentation on obesity, nutrition and physical activity
				• Discuss parents’ own eating and physical activity habits
				- What could be changed? What are the factors that hinder change?
				• Give homework
	Between sessions			• Homework for parents
				- Food registration
3	Individual session	45	12	• Discuss the group session
				• Discuss parents’ own eating and physical activity habits (based on the food registration)
				• Formulate goals together with parents concerning their healthy eating and physical activity
				• Discuss children’s eating habits, food choices
				• Formulate goals together with parents concerning their child’s healthy eating and physical activity
4	Individual session	30-45	18	• Motivate parents to continue to act as role models for their children concerning healthy eating and physical activity
				• Follow-up on the goals formulated during the 3rd session
				• Talk about how to get healthy eating and physical activity into everyday life (including day-care centres)
				• Reflect together with parents on child’s weight development
				• Formulate goals together with parents concerning their own and their child’s healthy eating and physical activity
5	Individual session	45	24	• Increase parental self-efficacy in succeeding with behaviour change
				• Motivate parents to continue healthy eating and physical activity
				• Follow-up on the goals formulated during the 4th session
				• Reflect with parents on child’s weight development
				• Discuss how parents can handle risk situations for unhealthy behaviours
				• Give homework
	Between sessions			• Homework for parents
				- Food registration
				• Printed form with questions to reflect upon before telephone session is sent to parents
1	Telephone conversation	15	30	• Discuss the printed form
				• Go through the homework from the 5th session
				• Follow up on parents’ progress on their own and their child’s goals
				• Continue motivational work
				• Encourage parents and highlight their successes
				• Support parents in solving problems concerning setting limits on the unhealthy behaviours of their children
6	Individual session	45	36	• Follow up parents’ progress on achieving their goals
				• Review all the goals of the intervention programme
				• Reflect with parents on child’s weight development
				• Continue motivational work
				• Increase parental self-efficacy in being a good role model and in helping their children with healthy behaviours
				• Discuss difficulties and hindering factors, and ways of addressing them
				• Introduce and set maintenance plan together with parents
	Between sessions			• Homework for parents
				- Food registration
				• Printed form with questions to reflect upon before telephone session is sent to parents
2	Telephone conversation	15	42	• Discuss the printed form
				• Follow up on parents’ progress on achieving their goals
				• Continue motivational work
				• Focus on parent’s confidence and emphasise parental autonomy
				• Follow up on the maintenance plan
7	Individual session	45	48	• Continue motivational work
				• Focus on parents’ confidence and highlight parental autonomy
				• Discuss the necessary conditions for maintaining the child’s healthy habits during childhood

### Adherence

The intervention and control nurses are invited to separate meetings twice a year with their local study coordinator. These meetings focus on maintaining high motivation for, and commitment to, good performance in accordance with the project plan, the detailed guidelines in the project instructions, and the intervention manual (intervention nurses only). Further, the local study coordinator monitors the performance of all intervention and control nurses in their geographical area twice a year by inspecting all relevant documents for completeness, e.g., goal formulation sheets, data on height and weight, data on time used in the sessions, and information on the adult(s) visiting the CHC with the child.

To measure intervention nurses’ adherence to the manual, a random sample of 100 families was drawn to assess completeness with regard to the content of the first goal formulation sheet to be filled in by the nurses during the third session (when the child is 12 months-old). The sample comprised 29 of the 43 intervention nurses (67%). According to the manual, CHC nurses should have a dialogue with their families concerning their goals on eating and PA habits. One rater scored completeness of goal setting formulation sheet in terms of content by using a pre-developed scoring schema.

As preparation for goal setting, parents are instructed to fill in a short food diary as homework before the third session and also before the telephone follow-up at 30 months of age of the child. The food diary is intended to raise parents’ awareness of existing family eating patterns, not to calculate food intake. Parents’ adherence to the intervention is measured by assessing the completeness of the food diaries of the random sample of 100 families (100 mothers, 83 fathers), described above. Additionally, data are collected on parents’ attendance at intervention sessions, as another indicator of parents’ adherence to the intervention.

### Measurements

At four years of age, the Body Mass Index (BMI) and waist circumference (WC) of the participating children and their mothers will be compared between the intervention group and the control group. Four-year-old children’s BMI and WC are the primary outcomes, and will be used as indicators of obesity status. CHC nurses weigh and measure the children on validated scales and stadiometers. Since BMI is rarely measured at exactly four years of age at CHCs, we will predict BMI at four years using a non-parametric regression method called kernel smoothing [27]. International definitions are used to classify children’s body sizes as underweight, normal weight, overweight, or obese [28].

Secondary outcomes are mothers’ BMI and WC, children’s and mothers’ PA and eating habits, and also parental self-efficacy (PSE). The eating habits of children and mothers are assessed with a FFQ that includes questions about habitual dietary intake of certain food items as well as intake of beverages, and is complemented by questions about regularity of breakfast eating. Parents record their children’s food intake at home but not the children’s intake during day-care. Mothers, on the other hand, record their complete dietary intake. The dietary outcome variables evaluated in the PRIMROSE study concern fruit, vegetables, fish, French fries, sugared drinks, discretionary calories, and the regularity of breakfast eating. The concept of discretionary calories was introduced by the Swedish National Food Agency, and is a collective name for products that have little nutritional value, but are high in energy, e.g., savoury snacks, sugared drinks, sweets, chocolate, pastries, cakes and ice cream.

The FFQ has been compared against an 8-day food diary (FD). Participants were a random sample of mothers and their preschool children, aged three or five years, not participating in the trial, but living in the same counties as those of the PRIMROSE study population. A total of 2400 mother-child pairs were invited to participate. The data were collected in 2008 during spring and autumn. The FD was performed at two occasions, corresponding to Wednesday–Saturday and Sunday–Wednesday The second 4-day FD was scheduled to be sent out approximately four weeks after the first 4-day FD and the FFQ two weeks after the second FD. The mean intake of the two Wednesdays was calculated before converting the intake of the 8-day FD into mean intake during an ordinary week of five weekdays and two weekend days. Mothers were instructed to record only the food that the child ate together with the family, but to record their own dietary intake in full. Mothers were also instructed to use measuring cups or a food template (distributed together with the FD), or to weigh the food to estimate intake. The relative validities of the dietary outcome variables are presented in the Results section. Spearman correlation coefficients with 95% confidence intervals were used to assess relationships between the frequencies of food intake reported in the FFQ and the 8-day FD.

Differences in PA between mothers in the intervention group and their counterparts in the control group are investigated using the Baecke questionnaire, which covers three dimensions of PA, sports activity, leisure activity and work activity, with response options ranging from 1 (lowest level of activity) to 5 (highest level of activity) [29]. The questionnaire has been shown to have good validity in comparison with energy expenditure measured by the doubly labelled water method [30]. Differences in PA between children in the intervention group and those in the control group are investigated by using an Actigraph GT3X + accelerometer for a period of one week, when children reach the age of four. Accelerometers provide objective measurements of PA and sedentary lifestyle [31,32], but, so far, there is limited research on suitable cut-off points between different levels of PA among preschool children. Up until now, the cut-off points suggested by Butte et al. [33] seem to be the most appropriate for predicting PA using tri-axial accelerometers; for preschool children: sedentary ≤ 100 counts per minute (cpm); light 101-3907 cpm; moderate/vigorous ≥ 3908 cpm [33]. Mothers’ PA is examined on enrolment and again when their children reach four years of age, while the children’s PA is examined at four years of age only. PSE is measured both at baseline and follow-up using the Parental Self-Efficacy for Promoting Healthy Physical Activity and Dietary Behaviors in Children Scale (PSEPAD), a 14-item scale that covers three domains relevant to early childhood obesity prevention [34]. On an 11-point Likert scale (0 = not at all – 11 = to a very high degree), parents rate the strengths of their efficacy beliefs concerning promoting healthy dietary and PA behaviours, and the setting of limits on unhealthy behaviours in their children.

At baseline and follow-up, mothers also respond to questions about their socio-economic and socio-demographic circumstances (e.g., country of birth, educational level, occupation, and housing).

### Follow-up of families

When the children turn four years-old, mothers receive an invitation to fill in a follow-up questionnaire regarding their own and their child’s eating and PA habits. This can be done in web-based or paper-based format. The questionnaire is delivered in conjunction with return of the accelerometer. In case of no response, families are reminded on three consecutive Mondays by postal mail. Families that cannot be reached by mail are called, or contacted via the short text message service (SMS).

Families who move to another CHC that is not participating in the trial are requested to provide follow-up data. The measurements are taken either at the new CHC or during home visits by trained research assistants.

### Replacement of intervention and control nurses

Whenever a nurse drops out of the study, the administrative head of the CHC should, according to the contract signed with the principal investigator, appoint a replacement nurse for the families concerned. Nurses replacing an intervention nurse follow the same five-day educational programme described above. However, nurses who are unable to attend this standard training programme are offered an alternative. They first meet with the central project coordinator for a general introduction and practical information on tasks and responsibilities, which is followed by a full day of MI training with the project’s senior psychologist. Thereafter, there is distance training, based on the intervention manual, educational movies and a presentation of the standard course, which is available on the project’s website (accessible only with a user-ID and password). The material concerns SCT, MI, and also nutrition and PA. Most importantly, four supervision sessions are offered, based on the nurses’ own MP3-recorded training sessions (as in the standard training programme described above).

### Statistical analysis

Based on variance estimates from similar Swedish data on 4-year-olds, we have estimated that the statistical power is approximately 90% to detect a mean BMI unit difference of 0.3 kg/m^2^ at four years of age. This assumes 410 children in the intervention group and 540 children in the control group (with group sizes based on mothers who filled in the baseline questionnaire in time, and excluding drop outs).

At four years of age, the BMI and WC of the participating children and their mothers will be compared between the intervention group and the control group, taking into account the cluster-randomised study design [35]. Primary analyses will include intention-to-treat analysis and per-protocol analysis.

For comparison of baseline characteristics (Tables 2, 3 and 4) we performed t-tests for continuous variables and χ^2^-tests for categorical variables, which were derived from un-adjusted linear and multinomial regression models with robust (sandwich) variance estimators [35], using Stata/IC 12.1 software (StataCorp, College Station, Texas, USA).

**Table 2:** Baseline characteristics of the mothers

	**Intervention (n = 489)**	**Control (n = 550)**	
	**% (n)/Mean (SD)**	**% (n)/Mean (SD)**	**p-value**
**Age**	30. 3 (5.1)	29.4 (5.0)	0.108
**Education**			0.306
Primary	2.7 (13)	2.9 (16)	
Secondary	30.2 (149)	37.1 (204)	
Post-secondary	66.8 (327)	59.7 (328)	
**Born in Sweden**	94.7 (463)	91.1 (501)	0.036
**Smoking**			0.904
Current	8.0 (39)	8.9 (49)	
Former Smoker	26.4 (129)	27.5 (151)	
Never Smoker	65.6 (321)	63.6 (350)	
**BMI**	24.5 (4.5)	24.7 (4.8)	0.494
**BMI categories**			0.959
Underweight	2.5 (12)	2.7 (15)	
Normal weight	60.8 (295)	59.3 (325)	
Overweight	24.1 (117)	24.5 (134)	
Obese	12.6 (61)	13.5 (74)	
**Waist circumference (cm)**	83.7 (11.3)	84.2 (11.6)	0.494
**General Health Status**			0.941
Very good	22.1 (108)	23.7 (130)	
Good	58.2 (284)	58.2 (319)	
Fairly good	17.2 (84)	15.9 (87)	
Bad	2.3 (11)	2.0 (11)	
Very bad	0.2 (1)	0.2 (1)	

**Table 3:** Baseline eating and physical activity habits of the mothers

	**Intervention (n = 489)**		**Control (n = 550)**		**p-value**
	**Mean (SD)**	**Median**	**Mean (SD)**	**Median**	
**Eating habits**					
Fruit (t/d)	1.4 (1.0)	1.0	1.2 (1.0)	1.0	0.071
Vegetables (t/d)	2.0 (1.2)	1.8	2.1 (1.5)	1.7	0.568
Fish (t/w)	1.7 (1.8)	1.4	1.6 (1.4)	1.4	0.509
French fries (t/m)	1.4 (1.8)	1.0	1.5 (1.9)	1.0	0.298
Sugared drinks* (t/w)	2.3 (3.3)	1.2	2.6 (3.6)	1.4	0.134
Discretionary calories** (t/w)	9.6 (5.9)	8.5	9.9 (6.3)	8.9	0.425
Having breakfast (t/w)	6.7 (1.1)	7.0	6.5 (1.3)	7.0	0.207
**Physical Activity**					
Work	2.8 (0.7)	2.9	2.9 (0.7)	3.0	0.207
Sport	2.6 (0.7)	2.5	2.5 (0.7)	2.5	0.341
Leisure	3.0 (0.7)	3.0	3.0 (0.6)	3.0	0.636

t = times, d = day, w = week, m = month. *Sugared drinks include soda with sugar, lemonade, and chocolate drinks; **Discretionary calories include savoury snacks, sugared drinks, sweets, chocolate, pastries, cake, and ice cream.

**Table 4:** Baseline characteristics of the children

	**Intervention (n = 485)**	**Control (n = 556)**	
	**Mean (SD)**	**Mean (SD)**	**p-value**
**3 months of age**			
BMI (kg/m^2^)	16.0 (1.4)	16.2 (1.4)	0.019
Height (cm)	61.2 (2.6)	61.3 (2.4)	0.494
Weight (kg)	6.0 (0.8)	6.1 (0.8)	0.027
**6 months**			
BMI	17.2 (1.5)	17.3 (1.5)	0.053
Height	67.5 (2.4)	67.8 (2.4)	0.337
Weight	7.8 (0.9)	8.0 (0.9)	0.01
**9 months**			
BMI	17.3 (1.4)	17.5 (1.4)	0.068
Height	72.1 (2.5)	72.3 (2.5)	0.024
Weight	9.0 (1.0)	9.2 (1.0)	0.01
**Mean age at baseline**	6.7 (1.1)	6.7 (1.0)	
BMI	17.2 (1.5)	17.4 (1.5)	0.077
Height	68.5 (2.9)	68.7 (3.0)	0.433
Weight	8.1 (1.0)	8.2 (1.1)	0.041
**Current feeding mode**			0.956
Breastfeeding only	9.6 (47)	8.6 (47)	
Part-time	50.5 (247)	49.9 (274)	
Not any longer	36.6 (179)	38.3 (210)	
Never	3.3 (16)	3.3 (18)	

### Cost-effectiveness analysis

A cost-effectiveness analysis (CEA) will be conducted from a health care (payer) perspective, and a cost-utility analysis (CUA) from a long-term societal perspective. In the CEA, we will estimate the direct costs that would be incurred by an agency, e.g., a county council, in implementing this type of intervention, by comparing the intervention with care as usual with regard to differences in costs (incremental costs) divided by differences in health effects. The outcome will be the incremental cost-effectiveness ratio (ICER), which represents the additional cost of a one BMI-unit lower body weight among children in the intervention group compared with the control group. The time consumption of CHC nurses and parents in the intervention and control groups for all visits to their CHC from the ages of nine months to four years is recorded prospectively. Indirect costs mainly consist of parents’ absence from work due to their participation in the intervention. BMI at the age of four years is fairly strongly associated with BMI in adolescence and later life [36-38]. Based on our own empirical data [39] and data from the literature on risks of morbidity and mortality, we will estimate future productivity losses, e.g., due to disability pension and preterm mortality, and additional societal costs for one additional year of life gained by the intervention compared with care as usual.

## Results

### Baseline characteristics

The baseline questionnaire was administered at CHCs after obtaining informed consent from the families (n = 1369). Of the mothers who consented, 489 in the intervention group completed the questionnaire before the first intervention session. Due to time delays, an additional 46 mothers filled in the questionnaire after their first intervention session, and have therefore not been considered in the current results. In the control group, 550 mothers filled in the questionnaire before their children were 10 months of age (included here), but an additional 87 mothers filled in the questionnaire at a later stage (not included here).

The baseline characteristics of the 1039 mothers (with 1053 children) are presented in Tables 2, 3 and 4. Differences in the number of children and mothers are due to mothers with twins or triplets and or mothers who answered the questionnaire in time but quit before we had chance to collect growth data. At baseline, the mothers had a mean age of 30.3 (SD = 5.1) years in the intervention group, and 29.4 (SD = 5.0) years in the control group. Slightly more mothers were born in Sweden in the intervention group (94.7%) than in the control group (91.1%). The proportion of mothers with post-secondary education was somewhat higher among mothers in the intervention group (66.8%) than among those in the control group (59.4%). The proportion of mothers with overweight or obesity at baseline was similar in the intervention group (36.7%) to that in the control group (38.0%) (Table 2). The baseline eating and PA patterns of the mothers are presented in Table 3. Overall, there were no significant differences with exception of country of birth between the intervention and control groups, indicating successful randomisation. At nine months of age, the children had a BMI of 17.3 (SD = 1.4) kg/m^2^ in the intervention group, and 17.5 (SD = 1.4) kg/m^2^ in the control group (Table 4).

### Relative validity of the FFQ

Of the 2400 invited families, 514 (21.5%) agreed to participate. Sixty-eight percent of the mothers who wanted to participate completed the first 4-day FD. The second 4-day FD, which was sent to those who completed the first, had a response rate of 78%. The FFQ was sent to those families who had completed at least one of the two FDs. The relative validation was carried out among 214 mother-child pairs that had completed both 4-day FDs and the FFQ (response rate: 8.9%). Half of the children in the sample were girls, and mean age in the sample was 4.0 (SD = 1.0) years. According to self-reports of height and weight, 12% of the children were either overweight or obese. The mothers had a mean age of 37.2 years (SD = 4.4), and a mean BMI (self-reported) of 23.3 kg/m^2^ (SD = 3.9), and were relatively highly educated (61% with 3 years or more of post-secondary education). A majority of the mothers (92%) were born in Sweden, and 19% were overweight or obese. The time period between the recording of the first and the second 4-day FD was on average 24 days (range 8-85), and between the second 4-day FD and the FFQ, 23 days (range 3-141).

The mean and median frequencies of intakes of food and beverages reported in the FFQ and the 8-day FD, for children and mothers, are presented in Table 5. The differences between mean frequency intake values according to the FFQ and FD show that the FFQ provided higher estimates than the FD for a majority of the food groups, with the exception of sugared drinks.

**Table 5:** Relative validity of the food frequency questionnaire (FFQ)

	**Food diary**			**FFQ**			**Food diary and FFQ**				
	**Mean**	**SD**	**Median**	**Mean**	**SD**	**Median**	**n**	**d†**	**95% CI**	**r**	**95% CI**
Children											
Fruits (t/d)	0.89	0.55	0.79	1.19	1.07	1.00	210	0.30	0.19; 0.42	0.42	0.31; 0.53
Vegetables (t/d)	0.74	0.38	0.71	1.83	1.11	1.57	212	1.08	0.96; 1.19	0.48	0.36; 0.58
Fish total (t/w)	1.11	1.06	1.00	1.65	1.68	1.47	211	0.54	0.33; 0.74	0.31	0.18; 0.42
French fries (t/m)	1.21	2.00	0.00	1.36	1.50	1.00	210	0.14	-0.12; 0.40	0.36	0.24; 0.48
Sugared drinks* (t/w)	2.80	2.65	2.00	2.41	2.90	1.50	211	-0.39	-0.65; -0.13	0.59	0.50; 0.67
Discretionary calories** (t/w)	8.38	4.39	8.00	9.16	5.79	8.05	212	0.75	0.18; 1.33	0.56	0.46; 0.65
Mothers											
Fruits (t/d)	1.15	0.75	1.07	1.63	1.09	1.43	213	0.48	0.37; 0.59	0.60	0.50; 0.68
Vegetables (t/d)	1.31	0.49	1.29	2.76	1.40	2.57	213	1.44	1.29; 1.59	0.32	0.19; 0.43
Fish total (t/w)	1.86	1.53	1.50	2.25	1.92	2.00	205	0.36	0.13; 0.60	0.41	0.28; 0.51
French fries (t/m)	0.99	1.91	0.00	1.23	1.54	1.00	211	0.23	0.00; 0.46	0.34	0.22; 0.46
Sugared drinks* (t/w)	1.43	1.89	1.00	1.33	1.94	0.75	209	-0.13	-0.37; 0.11	0.43	0.32; 0.54
Discretionary calories** (t/w)	8.29	4.29	8.00	8.51	5.60	7.42	198	0.19	-0.45; 0.83	0.52	0.41; 0.61

r = Spearman correlation, t = times, d = day, w = week, m = month, CI = confidence interval. *Sugared drinks include soda with sugar, lemonade, and chocolate drinks; **Discretionary calories include savoury snacks, sugared drinks, sweets, chocolate, pastries, cake, and ice cream. †Difference between mean FFQ and mean food diary.

The results show that there were strong correlations between the FD and the FFQ for mothers’ fruit intake. Moderately strong correlations were found for children’s fruit and vegetable intake, mothers’ and children’s intake of discretionary calories, mothers’ intake of fish, and mothers’ and children’s intake of sugared drinks. Weak correlations were seen for children’s fish intake, mothers’ and children’s intake of French fries, and mothers’ vegetable intake (Table 5). Graphical evaluation of Bland-Altman plots showed that most of the observations fell within the 95% limits of agreement (data not shown). In general, there was an increase in the differences between the results of the two methods as intake increased. While agreement between the two methods was fairly good at lower levels of intake of vegetables among both children and mothers, the differences increased with increasing vegetable intake, suggesting over-reporting in the FFQ compared with the FD at higher levels of intake. For both children’s and mother’s fruit intake, there was a systematic negative difference at lower intakes and a positive difference at higher intakes.

Mothers with normal weight, compared with overweight, tended to be more accurate in reporting of their own fruit intake (r = 0.67 vs. r = 0.35), their child’s fruit intake (r = 0.50 vs. r = 0.08), and their own intake of discretionary calories (r = 0.57 vs. r = 0.37). Mother’s with normal weight were also more accurate in reporting of their child’s fish intake (r = 0.29 vs r = 0.06). However, there were stronger relationships between the FD and the FFQ for maternal vegetable intake among mothers with low education compared with mothers with high education (r = 0.48 vs. 0.18).

## Discussion

The on-going population-based PRIMROSE trial, aimed at the primary prevention of childhood obesity from infancy to age 4, is conducted at CHCs responsible for the usual preventive child health services available free of charge to all families in Sweden. The manual-based PRIMROSE intervention, which is embedded in routine child health services, is conducted by ordinary CHC nurses who have attended an educational programme covering SCT and MI. We are not aware of any previous randomised trial that has investigated the effects on 4-year-old children’s BMI and WC of interventions using this theoretical framework and an evidence-based counselling method (MI) to influence parents’ and their children’s eating and PA behaviours.

Given that only 12.8% of eligible nurses participate in the PRIMROSE trial, there is the inherent possibility of bias in the selection of nurses from the general nurse population. When in 2008 and 2009 the rules for reimbursement of primary health care centres (including CHCs) changed, it became less profitable to spend time on health promotion and disease prevention. Although many CHC nurses expressed interest in participating, some were unable to do so, due to a heavy workload, and limited funding from the project to provide full compensation for the extra time needed to carry out the intervention. However, the participating nurses do not differ from all the CHC nurses employed in the study area with regard to tenure, training, and the number of children for whom they have responsibility.

The intervention has a specific design and structure, and reasonably good proficiency in Swedish is needed to benefit from its MI-based component. Accordingly, the PRIMROSE trial only includes Swedish-speaking families. Of the first-time mothers (age ≥ 18 years) with children born 2008-2010 in the general Swedish population, 19.4% were born outside Sweden. Previous studies indicate that foreign-born parents and their children tend to have a higher prevalence of overweight and obesity than their Swedish-born counterparts [40,41]. Not including individuals with insufficient skills in the Swedish language (n = 172) means that future results may not be representative of families that rather recently migrated to Sweden.

Only a few previous studies have investigated the effects of the counselling of parents with young children on dietary habits using validated dietary outcomes [15]. Furthermore, the evidence is often restricted to intakes of fruit and vegetables as outcomes. Other dietary outcomes (e.g., intakes of sugared drinks, fish, fast food, and unhealthy snacks) are often neglected. We have demonstrated that our short FFQ has good relative validity in estimating children’s intake of sugared drinks and mother’s fruit intake. We have also shown that the FFQ has acceptable relative validity regarding children’s intake of discretionary calories, and mother’s intakes of discretionary calories, fish and sugared drinks.

However, the relative validity is less strong for children’s intakes of fish and French fries, and also for mothers’ intakes of French fries and vegetables. Previous relative validation of FFQs, based on estimates of preschool children’s specific food intakes, show similar correlations to those presented here [42-46]. The FFQ for mothers has previously been validated against five non-consecutive 24-hour recalls in a sample of women and men with similar background characteristics, and from the same regions in Sweden as the populations in the validation and PRIMROSE studies [47]. The correlations show somewhat higher agreement between the FFQ and reference method in this previous validation than in the one employed for the current study. The test-retest reliability of the FFQ was also measured in this earlier study, and shows that intra-class correlation coefficients ranged from 0.43 to 0.64 for the outcome variables used in the PRIMROSE study.

Even though we recruited mothers and children from the same geographical area as that of the PRIMROSE study, the mothers in the validation sample had a higher education, and were slimmer and older, which may be due to a low response rate in the validation sample. The higher intakes of healthy foods and lower intakes of unhealthy foods, especially sugared drinks, among mothers in the validation study compared with mothers in the PRIMROSE study suggest that mothers in the validation sample were more health-conscious. Furthermore, the influence of background factors, such as level of education and weight status, on the relative validity of fruit and vegetable intake that was observed in the present validation study needs to be accounted for when evaluating the dietary outcomes. This is especially important for the children’s dietary outcomes, because they are only measured at follow-up. Few studies have investigated the relationships between background characteristics and relative validity in different food groups in such a young age group. However, previous studies have not found any difference due to education level or weight status [45,46].

## Conclusion

The PRIMROSE trial is conducted within regular Swedish CHC services, under normal working circumstances. CHCs are visited by almost all Swedish families, and CHC nurses from diverse geographical areas are participating. Our results may, therefore, become representative of the country as a whole. If the intervention is effective, the cost-effectiveness analysis will show the additional costs incurred by the health care system in gaining, for example, one additional unit lower BMI among children in the intervention group compared with those in the control group.

In March 2014, when this paper was submitted, study enrolment was complete, and 304 children (75%) had completed the intervention. A majority (n = 824, 72%) of all the participating children had started follow-up measurements. The data collection will be finished by the end of 2014.

## Abbreviations

BMI: Body mass index; CHC: Child health care centre; FD: Food diary; FFQ: Food frequency questionnaire; MI: Motivational interviewing; PA: Physical activity; PSE: Parental self-efficacy; RCT: Randomised controlled trial; SD: Standard deviation; SCT: Social cognitive theory; WC: Waist circumference.

## Competing interests

The authors declare that they have no conflicts of interest.

## Authors' contributions

ND and LH drafted the manuscript. FR conceived the study, and had main responsible for its design in collaboration with AG. FR had main responsibility for coordination of the study, and helped to draft the manuscript. AG, BB, LF and ES wrote the intervention manual and conducted the educational programme for nurses. BB, PT, LF, BH, MM, MB and MWi made substantial contributions to the study design. MWe contributed substantially to study coordination and data collection. CL, BH and ES were involved in the conception of the relative validation study, including development of the FFQ. LH, ESA, MWi and SW conducted the relative validation of the FFQ. PT carried out the statistical analysis. All authors have read, critically revised and approved the final manuscript.

## Pre-publication history

The pre-publication history for this paper can be accessed here:

http://www.biomedcentral.com/1471-2458/14/335/prepub
